# Role of the TRPV Channels in the Endoplasmic Reticulum Calcium Homeostasis

**DOI:** 10.3390/cells9020317

**Published:** 2020-01-28

**Authors:** Aurélien Haustrate, Natalia Prevarskaya, V’yacheslav Lehen’kyi

**Affiliations:** 1 Laboratory of Cell Physiology, INSERM U1003, Laboratory of Excellence Ion Channels Science and Therapeutics, Department of Biology, Faculty of Science and Technologies, University of Lille, 59650 Villeneuve d’Ascq, France; aurelien.haustrate@inserm.fr (A.H.); natacha.prevarskaya@univ-lille.fr (N.P.); 2 Univ. Lille, Inserm, U1003 – PHYCEL – Physiologie Cellulaire, F-59000 Lille, France

**Keywords:** TRPV channels, endoplasmic reticulum, calcium signaling

## Abstract

It has been widely established that transient receptor potential vanilloid (TRPV) channels play a crucial role in calcium homeostasis in mammalian cells. Modulation of TRPV channels activity can modify their physiological function leading to some diseases and disorders like neurodegeneration, pain, cancer, skin disorders, etc. It should be noted that, despite TRPV channels importance, our knowledge of the TRPV channels functions in cells is mostly limited to their plasma membrane location. However, some TRPV channels were shown to be expressed in the endoplasmic reticulum where their modulation by activators and/or inhibitors was demonstrated to be crucial for intracellular signaling. In this review, we have intended to summarize the poorly studied roles and functions of these channels in the endoplasmic reticulum.

## 1. Introduction: TRPV Channels Subfamily Overview

Functional TRPV channels are tetrameric complexes and can be both homo or hetero-tetrameric. They can be divided into two groups: TRPV1, TRPV2, TRPV3, and TRPV4 which are thermosensitive channels, and TRPV5 and TRPV6 channels as the second group. TRPVs are important therapeutic targets in view of their role in various disorders such as cancer, neurodegenerative disorders, and pain. They are generally highly selective for calcium (ranging from the ratio 3:1 for TRPV2 to 100:1 for TRPV6).

TRPV channels are expressed in many cell types and tissues and are located mostly on the plasma membrane. A number of studies have been performed as to the role of these channels on the plasma membrane proving that these channels play an important role in calcium homeostasis. Besides, some of them have been also shown to be located in the endoplasmic reticulum (ER). ER is a large calcium store in cells and is definitely involved in calcium-triggered pathways. 

In the current review, we focus on the role of the TRPV channels in the ER. Among the TRPV channels found in the ER, there are TRPV1, TRPV2, TRPV3, and TRPV4. TRPV5 and TRPV6 are highly selective calcium channels, which were not shown so far to be expressed in the ER.

## 2. TRPV1 Channel in ER 

TRP vanilloid-1 (TRPV1) is the first and the founding member of the TRPV subfamily and was initially cloned from the rat dorsal root ganglia (DRG) [1]. It can be activated by various stimuli such as temperature (>43 °C), acidic conditions as well as by capsaicin. It has long been recognized that TRPV1 is located on the plasma membrane, serving to non-selectively permeate calcium ions coming from the extracellular medium into the cytoplasm. Interestingly, the increasing evidence suggests that TRPV1 is also located intracellularly in various cell types such as neurons [2], myocytes [3], and cancer cells [4]. 

Quantification of calcium flux into TRPV1-overexpressing cells demonstrated that 85 to 90% of functional TRPV1 was expressed on the ER membrane [5]. TRPV1 activation was associated with the changes in the expression of several prototypical ER stress genes in lung cells. TRPV1 activation was shown to promote cytotoxicity via activation of EIF2αK3, phosphorylation of EIF2α, and expression of GADD153.

TRPV1 plays an important role in nociceptive neurons. Two vanilloid receptor pools were detected in these neurons: one on the plasma membrane and the other in the endoplasmic reticulum. A study by [6] has characterized this functional significance using calcium imaging in both stably transfected cells and dorsal root ganglion neurons. It has been demonstrated using both TRPV1 activators such as capsaicin or resiniferatoxin, and Ruthenium red to block plasma membrane-localized receptors, that endoplasmic reticulum pool is independent of the plasma membrane pool. Authors also demonstrated that the direct depletion of calcium via activation of the endoplasmic reticulum-localized TRPV1, triggered the store-operated calcium entry. 

Activation of TRPV1-expressing insect cells (Spodoptera frugiperda (Sf 9)) produced an increase in the cytosolic free Ca^2+^ concentration and, in the absence of extracellular Ca^2+^, there was still an increase in the cytosolic free Ca^2+^ suggesting that intracellular TRPV1 (and not plasma membrane-localized TRPV1) could play an important role in calcium signaling [7]. TRPV1 channel was shown to be localized in the ER using confocal imaging. Moreover, 2-APB has also blocked thapsigargin-induced Ba^2+^ influx, but not that induced by resiniferatoxin. These data suggest that TRPV1 forms agonist-sensitive channels in the ER, which once activated, releases Ca^2+^ from internal stores. However, they do not activate the endogenous store-operated Ca^2+^ entry.

More recently, it was shown that the ER of dorsal root ganglion neurons contains functional TRPV1 channels [8]. The sensitivity to capsaicin of ER-localized TRPV1 was smaller than the sensitivity of the plasma membrane-localized TRPV1. However, the precise TRPV1 function in the ER is still misunderstood.

In 2014, Wong et al., reported that ER calcium release by the *Drosophila* TRPV channel Inactive (Iav) controlled the synaptic growth, synaptic transmission and presynaptic signaling inside the cell, while contributing to the Ca^2+^ resting levels [9]. The authors genetically screened most Drosophila TRP channels for a potential synaptic role at the level of the glutamatergic larval neuromuscular junctions (NMJs) and have found that mutations in *iav* impaired the synaptic growth. On the contrary, the expression of the human TRPV1 in i*av*-deficient motor neurons rescued these defects. *Iav* was also shown to be localized in the ER [9]. Loss of Iav resulted in the decreased presynaptic resting [Ca^2+^]i, as well as in the diminished synaptic transmission and synaptic vesicle release probability.

Furthermore, the activation of ER-localized TRPV1 by endo-vanilloids was able to trigger the ATF3-dependent ER stress pathway, which was sufficient to mediate the death of high-grade astrocytomas [10].

**Figure 1:**
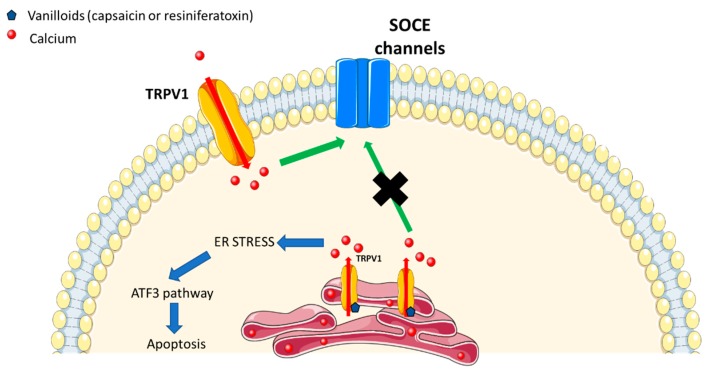
Endoplasmic reticulum-localized transient receptor potential vanilloid 1 (TRPV1) plays a different role than the plasma membrane-localized TRPV1 and can activate other calcium pathways.

All these data confirm the functional presence of TRPV1 as a Ca^2+^ release channel in the ER (Figure 1).

Capsaicin, the activator of TRPV1, increased the apoptosis in human nasopharyngeal carcinoma model by inducing ER stress. The use of capsaicin was shown to increase the levels of inositol-requiring 1 enzyme (IRE1), to produce reactive oxygen species (ROS), as well as the expression of GADD153 and GRP78. Furthermore, it increased calcium levels, induced cytochrome c release as well as caspase 3 and 9 activation (Figure 1) [10,11,12]. The selective cannabinoid type 1 (CB1) receptor agonist seemed to attenuate ER stress both in neurons and in the central nervous system. In fact, this agonist, ACEA, has also been reported to activate TRPV1 channels. While using ACEA and TRPV1 antagonists, the neuroprotective effect of ACEA was shown to be reversed, suggesting that TRPV1 is crucial in neuroprotective effect of ACEA against ER stress [13,14].

## 3. TRPV2 Channel in ER

Transient receptor potential vanilloid type 2, TRPV2, is a non-selective cation-permeable cation channel which is activated by heat (>52 °C), ligands such as cannabidiol [15], 2-APB [16], probenecid [17] as well as mechanical stresses. In most of the cells, a significant amount of TRPV2 is situated in the ER under unstimulated conditions. Upon stimulation of the cells using, for example, phosphatidylinositol 3-kinase-activating ligands, TRPV2 is translocated to the plasma membrane and functions thereon as a cation channel. Mechanical stress was shown to also induce the translocation of the TRPV2 channel to the plasma membrane. The TRPV2 expression was shown to be high in some cell types including neurons, neuroendocrine cells, innate immunity-involved immune cells, as well as in certain types of cancer cells. TRPV2 was shown to modulate various cellular functions in these cells [18,19,20].

TRPV2 channel is obviously expressed in various tissues including the central nervous system, neuroendocrine cells and epithelial cells in the kidney as well as in the liver [21]. IGF1 induced TRPV2 translocation towards the plasma membrane depending on the activity of the phosphatidylinositol (PI) 3-kinase [22]. Among six members of the TRPV family channels, the expression of only TRPV2 channel was detected in macrophages. TRPV2 channel is mainly localized in the ER [23]. In macrophages, a chemotactic peptide fMLP also induced the translocation of TRPV2 from ER to the plasma membrane. fMLP activated PI 3-kinase by a G protein-dependent mechanism which was sufficient to translocate TRPV2 to the plasma membrane [23].

In Duchenne muscular dystrophy (DMD) myotubes, the store-operated calcium entry (SOCE) was reported to be increased under the influence of calcium/PLC/PKC pathway. Similarly, α1-syntrophin regulation increased the TRPV2-dependent cation influx [24].

## 4. TRPV3 Channel in ER

It has been more than 15 years since TRPV3 has been discovered as a heat-sensitive but capsaicin-insensitive channel [25]. It can be activated by physiological temperatures (between 33 °C and 39 °C) and by selective both endogenous and exogenous compounds such as 2-Aminoethyl diphenylborinate 2-APB [26], diphenylboronic anhydride (DPBA), diphenyltetrahydrofuran (DPTHF), farnesyl pyrophosphate [27]. TRPV3 is widely expressed in physiological conditions, especially in the skin [28] and in the brain [29]. Its expression can be modified leading to disorders such as cancer [30,31]. 

It has been recently demonstrated using calcium imaging technique that TRPV3 is expressed in the endoplasmic reticulum of Embryonic Stem Cells (ESCs) [32]. In fact, when extracellular calcium is depleted, the cytosolic calcium can be increased using TRPV3 activators but not in the case when ER stores were already depleted. The authors have shown using trypan blue and MTT assays, a decrease in mESC proliferation when TRPV3 was activated. Cell cycle analysis revealed that TRPV3 activation arrested mESCs at G2 /M phase [32], suggesting its role in cell cycle control.

Recently, some mutations in TRPV3: G573A, G573S, G573C and W692G have been detected leading to such disorders like Olmsted Syndrome (OS) [33,34] as well as other skin disorders [35]. These mutants were mainly retained in the ER [36] leading to reduced surface localization.

## 5. TRPV4 Channel in ER

TRPV4 was identified as a mechanosensitive channel [37]. It is involved in the multiple physiologic functions (systemic osmotic pressure, sensory system, skin homeostasis, urinary system, hepatic), dysfunctions (neurological disorders), and diseases. It can be activated by numerous stimuli such as osmotic stress, temperature (>34 °C), and pharmacological compounds. In 2006, Arniges et al. identified five splice variants of TRPV4, sub-divided into two groups. The first group (TRPV4-A and TRPV4-D) was successfully processed in the ER and was targeted to the plasma membrane, while, the second group variants, (TRPV4-B, TRPV4-C, and TRPV4-E) were retained. All alternative spliced variants involved the deletions affecting the ankyrin domains except for TRPV4-D. In fact, ankyrin domains are essential for oligomerization of TRPV4 and lack of oligomerization was shown to be responsible for its accumulation in the ER [38]. The STIM1 protein was demonstrated to associate specifically with the C-terminal tail of TRPV4 to form a complex regulated through phosphorylation of serine824 of TRPV4 [39].

Other data showed the crucial role of C-terminus domain in oligomerization of TRPV4 in the ER [40]. In addition, the C-terminus may also interact with the N-terminus [41]. Wang et al. showed that OS-9, a ubiquitously expressed endoplasmic reticulum (ER)-associated protein, interacted with the cytosolic N-terminal tail of TRPV4. They have found that this protein can block TRPV4 output from the ER and consequently decrease its expression at the plasma membrane. OS-9 bound to monomers and immature TRPV4 variants to decrease polyubiquitination. In fact, OS-9 acted as protector of TRPV4 from ubiquitination and was a modulator of its transport [42].

Protease-activated receptor 2 (PAR2) is expressed on nociceptive neurons, and can induce the sensitization of TRPV4 channels, the activation of PLCβ, and the release of inositol trisphosphate (IP3), which stimulates the release of calcium from intracellular calcium stores [43].

Infrared radiations (carried out using an optical fiber) produced an increase in cytosolic calcium concentrations in spiral ganglion neurons which was mediated by the release from intracellular stores. When TRPV4 was blocked, the increase in the cytosolic calcium concentration was abolished suggesting that the activation of TRPV4 channels played a crucial role in the regulation of IR-induced calcium level elevations providing a new tool to deplete intracellular calcium pools [44].

In addition, it was shown that TRPV4, TRPC1, and TRPP2 can assemble to form a flow-sensitive heteromeric channel [45]. TRPP2 can protect cells from apoptosis while reducing calcium levels in the ER [46], suggesting a complex role of these channels in this mechanism.

## 6. TRPV5 and TRPV6 Channels

TRPV5 and TRPV6 channels are only highly selective calcium channels inside TRV subfamily. They can form either homo or heterotetramers. Physiologically, TRPV5 is mostly expressed in the kidney and TRPV6 is mostly expressed in the intestine. They were not shown to be expressed in the ER but they can indirectly modify their expression at the plasma membrane. In fact, when calcium is depleted from the ER, this depletion is recognized by STIM1 EF-hand domains. This recognition will permit STIM1 unfolding. In such a way, STIM1 can interact with SOCE channels. Calcium entry provided by SOCE channels will allow for the TRPV6 translocation towards the plasma membrane by engaging Annexin1/S100A11 complex in prostate cancer cells. TRPV5 and TRPV6 being highly calcium selective and constitutively active, may let a large amount of calcium to enter inside the cell to fill up the calcium pool in the ER. The Annexin1/S100A11 complex may vary depending on the tissue: Annexin1/S100A11 for the prostate cancer [47] and S100A10/Annexin 2 complex in the intestine and the kidney [48].

TRPV6 was also shown to be involved in the apoptosis resistance and proliferation in various cell types [49,50,51]. It is known that ER stress induced by thapsigargin increased the TRPV6 expression in human embryonic stem cell-derived cardiomyocytes. However, the TRPV6 inhibition had no effect on ER stress. Cleaved-ATF6α caused a significant increase in the TRPV6 protein levels suggesting that ATF6α pathway may activate TRPV6 expression under the ER stress. TRPV6 overexpression was shown to play a protective role against the ER stress in human embryonic stem cell-derived cardiomyocytes. In this case, TRPV6 would decrease JNK activation which is a regulator of the apoptosis induced by the ER stress [52].

## 7. Relationships between Channels

As an example of the relationship among TRV channel subfamily, odontoblasts have been suggested to contribute to the nociceptive sensation in the tooth via the expression of the transient receptor potential (TRP) channels. TRPV1, TRPV2, and TRPV3 channels were shown to be expressed in native human odontoblasts HODs. The relative intracellular distribution of these three channels was similar while TRPV1, TRPV2, and TRPV3 proteins were preferentially detected in both mitochondria and ER. Thus, HODs could play an important role in mediating pulp thermo-sensation due to the expression of these three TRPV channels. The difference in their relative intracellular distribution may suggest an important role in the sensing of the outer stimuli [53].

The relationships and/or the crosstalks between channels are still not clear and their interaction needs to be further investigated.

## 8. Conclusions

It should be noted that the precise data about the role and function of TRPV channels have not been established so far. Though the role of only one channel, TRPV1, has been studied so far, our knowledge of all of them is still very poor. Other TRPV channel candidates are likely to be good candidates to be studied, especially TRPV4, which colocalizes together with STIM1—a key regulator of calcium pools in the ER. Many studies still focus on the role of TRP channels on the plasma membrane and their role in the other organelles is poorly elucidated. New techniques can also help to study the further role of ER-localized TRPV channels including, but not limited to, intracellular patch clamp technique and intracellular calcium imaging. Thus, the relationship between TRPV channels and their interaction needs to be further investigated.
